# Telemonitoring in Cystic Fibrosis: A 4-year Assessment and Simulation for the Next 6 Years

**DOI:** 10.2196/ijmr.5196

**Published:** 2016-05-03

**Authors:** Irene Tagliente, Leopoldo Trieste, Terje Solvoll, Fabrizio Murgia, Sergio Bella

**Affiliations:** 1 Pediatric Hospital Scientific Direction, Research Area of Clinical-Healthcare and Management Innovations Pediatric Hospital Bambino Gesù Roma Italy; 2 Department of Human Social and Health Sciences, University of Cassino and Southern Lazio Cassino Italy; 3 Management Institute of Management, Scuola Superiore Sant’Anna Pisa Italy; 4 Centre for Integrated Care and Telemedicine Norwegian Centre for Integrated Care and Telemedicine University Hospital of North Norway Tromsø Norway; 5 Pediatric Medicine Department of Pediatric Medicine Pediatric Hospital Bambino Gesù Roma Italy

**Keywords:** cystic fibrosis, telemonitoring, cost effectiveness, follow-up, eHealth

## Abstract

**Background:**

Innovative technologies and informatics offer a wide range of services to health districts, doctors, nurses, and patients, and is changing the traditional concept of health care. In the last few years, the availability of portable devices, their easiness to transport and use, and the capability to collect and transmit various clinical data have resulted in the fast development of telemedicine. However, despite its potential impact in improving patient conditions, and its cost effectiveness reported in literature, telemedicine is not in daily practice.

**Objective:**

The aim of this study is to provide evidence of the positive impact of telemonitoring proving the sustainability of an application by sending spirometry outcomes from patients’ homes to the hospital doctors via the Internet, and from doctors to patients by an additional phone call solution.

**Methods:**

We examined collected data related to clinical improvement of patients with cystic fibrosis (CF). The patients were followed-up at home using telemonitoring for a period of 10 years, with the aims to prove the sustainability of the methodology (transmissions of spirometry from the patients' home to the doctors and feedback from the doctors to the patients by phone call from the hospital). We stored and analyzed all spirometry transmissions received, and tested the possible presence to decrease the costs between the standard clinical trial (only ambulatory visits) and standard clinical trial with telemonitoring for the follow-up of patients with CF (telemedicine). This was done through an economic analysis of the costs for patients followed at home by telemonitoring. We assessed four years of observation and a simulation of total long-term costs between 2010 and 2020.

**Results:**

We discovered a potential saving of €40,397.00 per patient for 10 years, actualized at €36,802.97 for the follow-up of all patients enrolled.

**Conclusions:**

The results from the study suggest that telemedicine can improve the health of patients with CF. It is a relatively cheap and potentially sustainable solution, compared to standard clinical trials. However, to establish and prove the long-term effectiveness and cost-effectiveness, more controlled psychological and behavioral studies are needed.

## Introduction

Innovative technologies and informatics offer a wide range of services to health districts, doctors, nurses, and patients, and is changing the traditional concept of health care. In the last few years, the availability of portable devices, its easiness to transport and use, and the capability to collect and transmit various clinical data have resulted in a fast development of telemedicine [1].

The use of portable devices for monitoring patients can find its application both within and out of hospitals [2]. With respect to the former, telemonitoring has the advantage of increasing efficiency in managing more patients, increasing patients’ safety, increasing quality of works, and reducing those opportunity costs (costs related to the fact that professionals spend a lot of time performing activities that do not need professional skills and competencies). That the standard of care is not able to reduce this effect still remains to be demonstrated without a real reorganization of competences and activities of health care professionals. Meanwhile, the use of devices able to remotely monitor and assist patients at home (in cases of temporary disease like rehabilitation after an accident and chronic diseases) has been of increasing importance for many decades. In recent years, telemonitoring and games have been introduced in this second field of application as valid alternatives to the standard of care, not only for monitoring patients’ progress but also motivating them and increasing adherence to treatment and telerehabilitation [3]. In both fields of application, literature and research are progressing together and research on electronic medical records (EMR) [4] has a natural ally in telemedicine.

However, despite its potential impact in improving patient conditions and its cost effectiveness reported in the literature, telemedicine is not in daily practice. The same is the case regarding the economic dimension and societal impact of telemedicine [5]. The lack of clear evidence is relevant for the low registering of telemedicine and telemonitoring diffusion (in hospital and home assistance cases) by the following five mechanisms. The first is reduced acceptability of information and communication technology (ICT) by the elderly. However, this barrier shows a decreasing trend; people in general and the elderly are more and more confident using ICT. The common idea that people over the age of 65 tend to be less interested in adopting new technologies for everyday problems is a myth. The second is the resistance by physicians and other health care professionals within and outside of hospitals who consider telemedicine and telemonitoring as a substitutive solution of their activities instead of tools for improving the quality and quantity of services delivered. The third is the fact that telemedicine modifies medical information exchanges between health care producers probably by increasing coordination problems. The fourth is the fact that telemedicine and telemonitoring can be considered good solutions for increasing the quality of life for young people (eg, reducing productivity losses), but it can be a poor solution for the elderly that live alone and need more human relations [6]. The fifth mechanism is increasing privacy problems and the related resistance of transferring more information on patient conditions over a long period of time [6,7]. However, these problems seem theoretically bigger than they actually are.

Our work has the objective to add evidence of the positive impact of telemonitoring and to provide an economic perspective of its usefulness in the second field of application: the follow-up of patients with chronic diseases at home with the direct consequence of lowering periodical and emergency hospital visits.

This study is focuses on the follow-up of patients with cystic fibrosis (CF) using telemedicine. CF is characterized by progressive lung destruction caused by the obstruction of the airway due to dehydrated thickened secretions. Obstructed airways results in endobronchial infection and an exaggerated inflammatory response leading to the development of bronchiectasis and progressive obstructive airways diseases [8]. For these patients, spirometry shows a reduction in forced expiratory volume in the first second (FEV1), and in forced current volume (FVC) (around 2% of the expected yearly value) [9].

Prevention and control of lung infections is one of the main objectives of therapy for patients with CF with the aim of reducing the progressive decline of pulmonary function [10]. Distance monitoring of lung parameters has been used in the follow-up of patients with CF in the Cystic Fibrosis Centre of Pediatric Hospital Bambino Gesù in Rome since 2001. A statistically significant reduction in hospital admissions and an over-time tendency towards a better stability of respiratory function was observed. [11].

In the present study we examined economic data related to the activities of telemonitoring for patients with CF followed at home for a period of 10 years, with the aim to better understand the evolution of clinical trends and costs over time. Here, we attempted to quantify the costs of telemonitoring (application of telemedicine) in the follow-up of the patients.

## Methods

The study was conduced in the Cystic Fibrosis Centre of Paediatric Hospital Bambino Gesù in Rome, Italy. The Cystic Fibrosis Centre of Pediatric Hospital Bambino Gesù is recognized as the national support center for CF; the center follows 280 patients from all over Italy each year. Since 2001, 57 patients were enrolled using telemedicine as follow-up, and 41 of them are still under telemedicine assistance (telemonitoring). The telemedicine CF team consists of three doctors, two respiratory physiotherapists, a nutritionist, two psychologists, two doctors in microbiology, and a biomedical engineer.

We analyzed data collected from 2010 to 2014, and then we simulated the saving of costs for the next 6 years. We enrolled 39 patients, but for the economical evaluation, we included only a subgroup of 25 patients. These 25 patients are those we were able to track for real costs, that is limited to the national health system (direct health), for the follow-up of patients with CF with telemonitoring assistance. Patients included in the telemonitoring program are still followed-up and treated using the standard protocols for follow-up. These protocols are the same for the patients not included in the telemedicine program (control group) [12].

**Figure 1:**
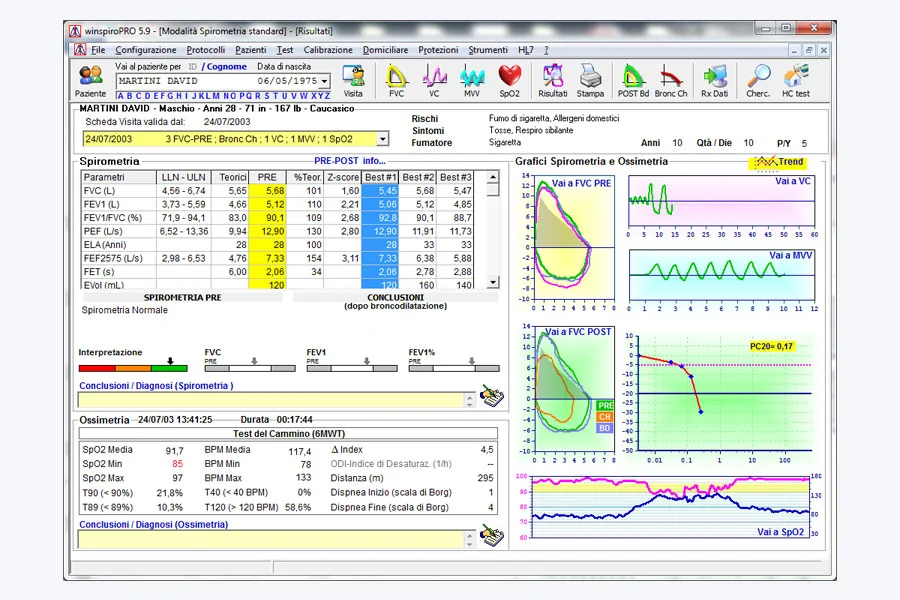
Screenshot of the software interface.

We used MIR-Spirotel instrumentation, which collects and remotely transmits data from a spirometer and overnight pulse oximetry. The method was described and discussed in a previous study [8]. The doctor prescribed each patient a spirometer after the diagnosis, which was delivered by the local dealer of regional health system (cost not provided for control group). Patients and parents were trained by physicians on the use of the device and how to send data to the Pediatric Hospital Bambino Gesù. Patients sent information twice a week. Data interpretation was performed using WinspiroPRO (Figure 1). The software was provided for free by the hospital’s spirometer dealer, and can display the spirometry curves and the main parameters FEV1, FVC, peak expiratory flow (PEF), and forced expiratory flow (FEF, 25-75%).

Further, the anamnestic data and graphs obtained were discussed in a meeting between CF doctors for an overall evaluation of clinical significance to decide on the possible therapeutic action. Patients showing a significant decrease of peripheral capillary oxygen saturation (SpO2) and/or FEV1 were invited to ambulatory visits.

Additional maintenance costs of instrumentations are not included on rental costs because the rental dealers absolved it. The annual median value of FEV1 was calculated using "before the month median value" for each patient and after we meshed the data. We also kept track of other indicators related to the interaction between doctors and patients with possible economic interest. For the economical evaluation, it was only possible to collect and keep track of all cost entries incurred for 64% (25/39) of the enrolled patients with CF. The type of data collected is shown in Textbox 1.

**Collected data.**

Data

- Hospitalization costs
  - Day hospital
  - Ambulatory visit
  - Intravenous therapy at home (minimum 21 days)
  - Oral therapy at home (minimum 21 days)
- Instrumentation costs
  - Monthly fee for instrumentation Spirotel
  - Rental of instrument for 25 pieces
- Doctors working days

We also considered all the cost entries for the Italian National Health System in the follow-up of patients with CF without telemonitoring assistance (control group), using historical data (hospital’s annual reports). For the analysis, we included 25 patients (17 female, 8 male), compliant and less compliant with the protocols, but adherent to the study. For the saving projection, we performed an analysis of the costs. The starting costs during the first year of telemonitoring assistance compared with the cost incurred to the follow-up of patients with CF with traditional trials (control group) are presented in Table 2.

For the economic evaluation, we analyzed 4-year costs using the following equations:

Total costs = net cost + rental of instruments (1)

Total savings = savings of vacant beds + saving of working days recovery (2)

Net cost = total costs – total savings (3)

Actual saving = theoretical cost – actual cost (4)

Here, "savings of vacant beds" refers to saving costs obtained for lower days of hospitalization compared with hospitalization day for the control group, and "saving of working days recovery" is defined as saving costs obtained for lower hours of works for visits of patients with CF under telemedicine protocol, compared to hours of works for visits of patients with CF in the control group. For "theoretical cost" we made a simulation of costs incurred for the follow-up of patients in the control group. Having different cost items, it was only possible to make a simulation thanks to the expertise of the doctors, in addition to envision diagnosis-related group (DRG) costs (Table 1). We assumed that these reimbursements will not change during the period of simulation.

Based on these results, we made three different simulations of the long run cost: 2010-2020 for two groups, and then calculated their differences. Analysis started including the top four most compliant patients previously enrolled in the telemedicine protocol (Table 2). Therefore, the lowest compliant patients have been enrolled. Finally, simulation involved all the 25 patients previously enrolled in the telemedicine protocol (Table 3).

**Table 1:** Starting costs for follow-up of 25 patients with and without telemonitoring during the first year.

Group		Cost, €
**Control group (without telemonitoring) costs (hospitilization and home treatment), n=25**		156,085.00
**Study group (telemonitoring)**		116,139.81
**Cost of services provided by the National Health Service**		
	Equipment rental (25 pieces)	126,000.00
	Total debit	242,139.81
	Additional revenue for the use of beds	123,019.00
	Recovery of working days	9284.00
	Total credit	132,303.00
	Current costs (debit-credit	109,836,81
**Budget**		
	Saving with telemedicine	46,248.19
	Savings cost on first year per patient of follow-up with telemonitoring assistance	1986.42

**Table 2:** Saving projection including only the four must compliant patients enrolled, until 2020, at 2015 prices (base year).

Year	Saving, €	Average consumer price index (CPI) inflation^a^, %	CPI correct saving, €
2010	5351.00	n/a	5748.70
2011	8090.00	2.78	8456.19
2012	16,038.50	3.04	16,269.88
2013	21,382.25	1.22	21,429.28
2014	26,726.00	0.24	26,720.65
2015	32,069.75	-0.02	32,069.75
2016	37,413.50	-0.02	37,420.98
2017	42,757.25	-0.02	42,774.36
2018	48,101.00	-0.02	48,129.87
2019	54,444.75	-0.02	54,488.33
2020	58,788.50	-0.02	58,847.32

^a^Source is the Worldwide Inflation Data website [13].

**Table 3:** Savings projection for all 25 patients enrolled, until 2020, at 2015 prices (base year).

Year	Saving, €	Saving projection at 2015 prices, €
2010	1849.00	1986.42
2011	2030.00	2121.89
2012	6293.00	6383.79
2013	10,556.00	10,579.22
2014	14,819.00	14,816.04
2015	19,089.00	19,089.00
2016	23,345.00	23,349.67
2017	27,608.00	27,619.05
2018	31,871.00	31,890.13
2019	36,134.00	36,162.92
2020	40,397.00	40,437.42

Using 2015 as base year, saving actualization was based on the previous rates for saving before 2015 and the inflation rate of 2015 for each of the simulated savings from 2015-2020. The 10-year total saving was obtained at the current prices. Italian average inflation rates based on the consumer price index (CPI) for the years 2010-2015 were obtained from the Worldwide Inflation Data website [13].

**Figure 2:**
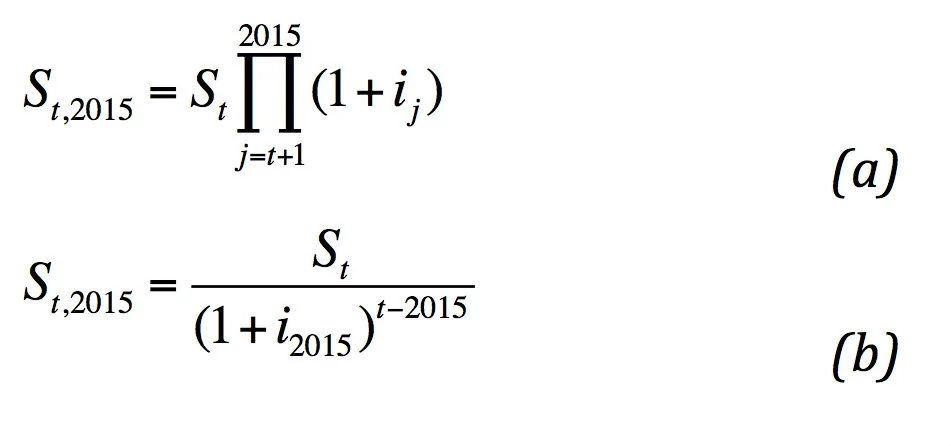
Economic equations used in the calculations. Equation (a) is the actualization of saving st (t<2015), and equation (b) is the actualization of saving st (t>2015).

The analysis was performed making three different simulations, with the economic equation, according to the number and type of patients included in the study, for each year under observation. Based on these results, we made a simulation of the long-term cost until 2020 for two groups, and then calculated the difference (Figure 2).

## Results

In 10 years of telemonitoring assistance, Dr Bella’s team followed a total of 39 patients. During the years of observation, we received 6647 spirometry transmissions. Some patients were excluded from the study for the absence of transmission, transmission problems of the oldest technology, or for death. With respect to the economic perspective, by tracking the costs of the follow-up of 25 patients with CF (compliant and less compliant with the protocol) with telemonitoring for 4 years, we show a difference between the cost incurred to the follow-up in compliance and non-compliance patients, as mentioned for diabetic tetrahydrocannabinol (THC) [10,11,14-17].

We found an increased cost saving, year by year, for prompt hospitalization with lower acute illness levels, before the incurrence of symptoms. After the second year of observation we already discovered decreased costs compared with cost incurred for the follow-up of patients with CF compared with traditional trials. In the economic analysis, including all patients enrolled, we observed a stabilization of the saving cost equal to €4263.00 per patient after the second year (Table 3).

The health service component done by the hospital to the patients with CF under telemonitoring, and savings cost compared with cost incurred for the follow-up of CF with traditional trials at 2015 prices are shown in Table 4.

**Table 4:** Health service component done by the hospital and savings cost compared with cost incurred for the follow-up of years 3 and 4 of the observation period at 2015 prices.

Health service component done by the hospital	2010	2011	2012
Day hospital, n	3	15	30
Ambulatory visits, n	0	0	4
Hospitalizations, n	12	15	12
No services, n	5	2	2
Total saving costs compared to traditional trials, €	1986.42/patient	2121.89/patient	6383.79/patient

Based on the years of accurate observation, we did a simulation of long-term saving from 2010-2020, adjusted by the inflation rate at 2015 price. This shows a saving of €40,437.42 per patient for the follow-up of all patients enrolled (Table 3). We made the same simulation including only the four most compliant patients, hoping to have a higher adherence with the treatment in the future [11]. From this simulation, as shown in Table 2 , we found a saving of €58,788.50 per patient for 10 years, €58,847.32 per patient at 2015 prices (the base year characterized by deflation instead of inflation). However, since price changes are not constant among years, it is necessary to update costs and saving once inflation rates will be available.

## Discussion

### Principal Findings

Here, we analyzed the quality and quantity of information transmitted and costs and/or savings of health resources related to telemonitoring in CF within a period of 4 years, and made a projection of costs for the next 6 years. The assessed potential saving of adopting a telemonitoring solution is relevant for a disease characterized by high costs for long-term treatments and follow-up. The adopted approach could be one of the first contributions in the field and also be used as a basis for analogous studies in different fields and diseases.

We found a decrease of costs incurred to the follow-up of compliant and non-compliant patients, as found in a previous study for telemonitoring of patients with diabetes [10]. We also found a significant difference between the three simulations and a greater decrease in cost with the more compliant patients and a reduction of savings in the patients with lower compliance to the telemedicine protocol. We show a similar trend with an exponential reduction of cost after the second year of follow-up with the telemonitoring protocols. The study shows an increase of cost saving incurred to the follow-up of compliant versus non-compliant patients, consistent with the literature of telemonitoring of patients with diabetes [15]. Data are encouraging with regard to the possibility of adopting telemonitoring and telemedicine solutions, offering a potentially more effective and cost-effective alternative to the current homecare assistance.

The approach is conservative since it does not take into account additional cost saving related to the improvement of therapy adherence induced by the use of technology. The progressive increasing adherence to treatment means a better overall use of the method.

With respect to the frequency of transmissions, we recommend to our patients a variable interval depending on the clinical condition: a minimum of twice per week, on average. We expect the optimal adherence to treatment to be 100% for two transmissions per week over a period of 5 working days [15]. Clinical practice shows a constant and progressive increase of treatment's adherence to achieve, in practice, a doubling of the values during the period under review.

Correlations between better therapy compliance for patients enrolled in telemonitoring, the increasing number of physiological parameters registered, and the lower costs for both national and international health systems suggest a technology-embedded solution for improving the quality of life of chronically ill patients.

These preliminary results have some implications for both the academy (from the medical and health economics perspectives), and the management of changes in activities of the health professionals involved.

This work provides an economic perspective of the assessment of telemonitoring, beyond the effectiveness and performance of the technology. With respect to the telemedicine literature, cost-effectiveness analyses are usually absent or the quality of analysis is very low [11]. Although a cost-effectiveness analysis should be the best approach in proving the utility of adopting telemonitoring, our work starts the process toward a cost-effectiveness analysis to focus on the national health care system perspective. However, this is the first step toward a simulation of the potential saving and the budget impact positive change induced by adopting technology in the follow-up of patients with CF.

### Benefits of Investing in Telemonitoring

The potential impact of the study results on current medical practices and management of the disease can be summarized in stressing the importance of telemonitoring inducing health care professionals and operators to invest in it. Investing in telemonitoring has the potentiality to produce benefits for patients, the national health care system, as well as the industrial sectors. Five benefits of investing in telemonitoring are described here. The first is improvements in the efficiency in managing different patients with respect to the current practice. The second are improvements in the quality of work of professionals (ie, reduction of stress and reduction of time spent in low value activities that can be performed by using telemonitoring). As a consequence, a reduction of opportunity costs for low-value routines and activities that are still performed by human beings is expected. The third are increases in the quantity and/or quality of available information from or to the patients and health care professionals, at lower costs and in reduced time. If integrated into a communication network, stakeholders and operators like patients, caregivers, health policy decision makers, governments, and public institutions can benefit by the network externalities produced by use of telemonitoring. In effect, the utility of adopting an additional telemonitoring device depends to the number of previous systems adopted. The virtuous cycle induced by adopting telemonitoring passes from an increasing adoption of telemonitoring devices to more available information (here data management and processing under the normative constraints related to patients’ privacy are mandatory) including (1) a deeper knowledge of cases that produce a better knowledge of the disease; (2) the increase of quality of individual care and assistance offered to singular patients; (3) the increasing demand of telemonitoring devices; (4) more competition in the market for heath device; (5) the improvement of technology performance and/or reduction of devices’ prices; and (6) the increasing utility of adopting new devices. The fourth benefit is turning negative into positive expectations among the health technology producers. Positive expectations induce more investment and new technological solutions sustained by the supply side of the market, reducing the risk of medium and long-run service and monitoring discontinuities. There is no health care sustainability without health technology sustainability, and vice versa. Finally, the fifth benefit is producing relatively cheaper and efficient technological solutions that should be considered the first.

### Limitations

One limitation is that we only considered direct health cost (ie, direct non-health and indirect saving costs for patients and their family) and indirect costs such as saving travel cost, saving working days, and saving school days have not been taken into account. A second limitation is that the simulation does not consider confidence intervals (ie, variance of costs and/or savings among patients) because costs are based on hospital tariffs rather than a micro-costing approach that may assess different costs for singular treatments and observations; costs of technology and a micro-costing approach should also be included considering different reimbursement scenarios. A micro-costing approach seems the best approach since up to now, telemonitoring or telemedicine solutions are not included in the Essential Levels of Assistance (LEA) provided by the Italian National Healthcare System (NHS), and there is not a DRG-based reimbursement. Adoption of telemonitoring still depends on individual cases and on voluntarily resources made available by the local health authorities. Because of its high potentiality in terms of medium and long-run saving from the societal perspective (the potential saving for the NHS perspective has been confirmed in the current study), we made a simulation of costs with actualization without taking under consideration the evolution of the disease as incurrence of not predictable comorbidity. In this, it is possible that during 10 years of monitoring some patients die from the disease or incurred co-morbidities. The increasing number of patients enrolled could increase the possibility that some patients’ expected lifespan will increase with a relative higher number of hospitalization for tests and related antibiotic treatments. This is especially true for the non-compliance patients with increases of per patient direct and indirect costs due to an increasing of working days lost for both patients and their caregivers.

For assessing the cost-effectiveness of telemonitoring in chronic disease, the best way for modeling the evolution of disease and the managerial perspective could share a discrete event model approach [18], rather than the usually adopted Markov models [19]. If the scope is to take into account the organizational implications of the disease management involving interacting agent, agent-based model simulations [20] should be implemented.

### Future Work

To track the impact of new technology with higher performance and be able to send the transmission directly from the device with mobile (SIM), and with the dissemination of broadband on the adherence of telemonitoring, future studies have to be implemented. We think that the constant evolution of technologies can help doctors to incentivize the patients on a better adherence to telemonitoring and self-management of their respective disease. At the same time, new studies are needed to track all costs incurred for the NHS for the follow-up of patients with or without telemonitoring assistance.

In addition, it could be of some interest to adopt a micro-costing approach for assessing the direct health costs and direct non-health costs. We expect a transportation cost-saving in using telemedicine and a reduction of time by formal and informal caregivers during their leisure time spent for assisting patients induced by the use of the technological solution to track the indirect cost (ie, productivity loss) incurred by the patients and their parents during the two different kinds of follow-up.

A comparison among different technology performances in terms of acceptability, effects, and costs could be investigated. This will allow the definition of requirements for new technology diffusion and adoption that is not related to technology only. Introducing new technology always has impact on organizations and decision-making. [21]. Organization, including activity, role, decision making, and interaction changes of current professional operators and patients and their relatives induced by telemedicine solutions should be investigated in more details. It is not only a necessary and very ambitious objective for an updated version of the current study, but a general need for proving the cost-effectiveness of telemedicine, and increasing its actual adoption for years to come.

### Conclusion

The current analysis reports a feasibility case study on adopting telemonitoring for CF follow-up. Patients included in this study report are still followed and treated with the usual protocols of follow-up, similar to those who do not practice [17].

The first results of this work have been encouraging. In a previous study we found a statistically significant reduction in hospital admissions and a tendency over-time towards a better stability of the respiratory function [22].

The equipment, a mobile phone with Skype connection, was used to keep in touch with the patients. The percentage of the successful calls appears to have improved over-time, but the mobile phone, in our opinion, continues to be valuable but not always completely reliable.

This study focuses on a preliminary simulation of the economic impact of telemedicine in terms of expected saving. From an economic perspective, health resource saving for the NHS is confirmed, supporting an economically viable method and/or trial. The increase in the calculated savings compared to our previous study indicates, in our opinion, a better efficiency of follow-up.

The advantage, in terms of quality of life for the patients, remains due to an at-home tool that allows patients to more easily stay in contact with the CF center. Expected positive contributions of the technology in supporting patients located in rural areas, to interact and transfer information to the hospital without a connection problem, could be possible with a SIM connection. We expect a better compliance of patients with a related better management of the CF disease, and reduction of costs incurred by the NHS for the onset of co-morbidity.

## Abbreviations

CF: cystic fibrosis
CPI: consumer price index
DRG: diagnosis-related group
FEVI: forced expiratory volume in the first second
FVC: forced current volume
ICT: information and communication technology
NHS: National Healthcare System
SIM: subscriber identity module
